# Winter Wheat Vernalization Alleles and Freezing Tolerance at the Seedling and Jointing Stages

**DOI:** 10.3390/plants14091350

**Published:** 2025-04-30

**Authors:** Fangfang Liu, Wenxin Cao, Qiqi Zhang, Yao Li, Heng Zhou, Yingxiu Wan

**Affiliations:** 1 Crop Research Institute, Anhui Academy of Agricultural Sciences, Hefei 230001, China; liuff0510@163.com (F.L.); cwxchina@aliyun.com (W.C.); zhqq1982@126.com (Q.Z.); liyaohefei0551@163.com (Y.L.); 19016101618@163.com (H.Z.); 2 Anhui Key Laboratory of Crop Quality Improvement, Hefei 230031, China

**Keywords:** winter wheat, vernalization gene, freezing tolerance, seedling stage, jointing stage

## Abstract

This study explores the relationship between allelic variation of the vernalization genes (*VRN*) and the freezing tolerance at the seedling and jointing stages of winter wheat growth. It provides a basis for molecular marker development for freezing tolerance breeding of winter wheat. A total of 435 wheat accessions were used to identify and evaluate the freezing tolerance at the seedling stage using field tests, while 192 wheat accessions were used to evaluate the freezing tolerance at the jointing stage in climate chamber tests. The *VRN* genes of the wheat accessions were detected using allele-specific markers of the *VRN-A1*, *VRN-B1*, *VRN-D1* and *VRN-B3* loci, and the relationship between *VRN* genotype and freezing tolerance at the two developmental stages was tested. There were significant differences in freezing tolerance between the wheat accessions. Assessing the freezing tolerance of 52 wheat accessions at both the seedling and jointing stages revealed no significant correlation between tolerance at these two stages. The genotypic analysis found that *Vrn-D1* was the most frequent dominant allele in winter wheat, while no accession contained the dominant alleles *Vrn-A1* and *Vrn-B3*. Notably, freezing tolerance showed stage-specific genetic regulation; seedling-stage freezing tolerance strongly correlated with vernalization gene allelic combinations (*p* < 0.05), whereas jointing-stage freezing tolerance exhibited no such association. The presence of all recessive alleles *vrn-A1*, *vrn-B1*, *vrn-D1* and *vrn-B3* was required for strong seedling-stage freezing tolerance. The *VRN-D1* marker was effective for screening freezing tolerance materials under the premise that *vrn-A1* and *vrn-B1* alleles are recessive at winter wheat seedling stage.

## 1. Introduction

Wheat is one of the most important food crops, providing approximately 20% of the calories and protein for human nutrition. China is one of the two largest wheat producers and consumers in the world, accounting for 17% of global production and 16% of consumption. A high and stable wheat production is important in ensuring national food security [1]. Wheat is mainly grown in temperate climates. Under the intensification of climatic instability, low-temperature freezing damage occurs frequently, and has become a major meteorological factor that affects wheat’s safe production.

Freezing stress can inhibit various physiological and biochemical activities in wheat, including water use, cell membrane stability, photosynthesis, secondary metabolite synthesis, and plant hormone content [2]. In wheat production, freezing stresses are generally classified into two categories that are distinct in terms of phenology: winter freezing damage, which occurs at the seedling stage and impairs seedling establishment and vegetative growth, and spring freezing damage, which takes place from the jointing stage to the heading stage and disrupts reproductive processes. Both types of freezing damage can lead to reduced yield and lower grain quality [3,4,5]. The jointing stage (the anther connective tissue formation phase, ACFP) is widely recognized as the most suitable period for identifying the spring freezing tolerance of wheat. During this stage, the wheat stems are tender. When exposed to temperatures below 0 °C, it is extremely likely to cause the death of the main stem and large tillers. The Yellow and Huai Valley Wheat Region is the largest wheat-producing area in China. Frequent occurrence of jointing-stage freezing damage in this region causes a substantial reduction in final grain yield by 30–50% in severe cases, affecting nearly 42% of wheat sown areas. Therefore, it is important to study the jointing-stage freezing tolerance.

The freezing tolerance in wheat is a complex quantitative trait, and has been extensively studied. There are two evolutionary adaptive mechanisms: cold acclimation and low temperature vernalization enable wheat to resist cold at the seedling stage. Cold acclimation involves a series of accumulative physiological and biochemical processes that enhance tissue cold tolerance, mediated by various cold-responsive genes including *COR* (cold-regulated), *LTI78* (low-temperature induced 78), and *LEA* (Late embryogenesis abundant) genes [6]. Low temperature vernalization is the physiological requirement for the transition of winter wheat from vegetative to reproductive growth [7,8], and is also an adaptation for wheat to avoid freezing damage to reproductive organs. Vernalization response is determined by vernalization genes. Allelic variation at four *VRN* loci (*VRN1*, *VRN2*, *VRN3*, and *VRN4*) is well characterized. Winter habit is a recessive trait and the dominant *Vrn* alleles function in circumventing or reducing vernalization requirement. *VRN1*, which plays the most important role in vernalization, encodes a MADS-box family protein homologous to Arabidopsis *APETALA1* that acts as a floral activator and is expressed in leaves and shoot apical meristem [9]. *VRN2* inhibits flowering by regulating the expression of *VRN1* and *VRN3* [10]. *VRN3* is an orthologue of *Arabidopsis* flowering factor *FLOWER LOCUS T* (*FT*), and its presence leads to early flowering [11]. *VRN4* regulates the vernalization process of wheat by interacting with *VRN1* and *VRN3* [12]. *VRN1* and *VRN3* promote flowering and circumvent or reduce the requirement for long-term low temperatures to induce vernalization. There are three *VRN1* loci in wheat: *VRN-A1*, *VRN-B1*, and *VRN-D1* located in chromosome arms 5AL, 5BL, and 5DL, respectively [13]. *VRN-A1* is the most sensitive to temperature and has epistatic effects on *VRN-B1* and *VRN-D1* [14]. The *VRN-B3* loci are located on group seven chromosomes [11].

Numerous studies conducted to dissect the genetic architecture of freezing tolerance have highlighted the importance of *VRN* loci in freezing tolerance. The presence of a dominant *Vrn1* allele significantly reduces freezing tolerance. Accessions with dominant alleles at two or three *VRN1* loci generally have weak freezing tolerance, whereas genotypes with recessive alleles at all three loci have strong freezing tolerance [15,16,17]. The *VRN1* gene may enhance the freezing tolerance of wheat by interacting with cold-regulated genes [18]. Genes *Fr-A1*, *Fr-B1* and *Fr-D1* for frost tolerance are located at or near the *VRN1* loci in homeologous group five chromosomes [19,20]. *FR1* and *VRN1* synergistically regulate the expression of *Cor* regulated by *CBF* transcription factors that can enhance the freezing tolerance of wheat [21]. Transcript analysis showed that *VRN1* alleles directly regulate *CBF* genes and repress their expression, thereby reducing freezing tolerance during reproductive growth [22,23,24]. The recessive allele *vrn-A1* increases freezing tolerance 2.1-and 2.4-fold in both winter and spring wheat compared to the dominant allele *Vrn-A1* [25]. Futhermore, the copy number of *vrn-A1* also influences the freezing tolerance of hexaploid wheat [26]. Spring freezing tolerance also shows complex associations with *VRN1*, where *Vrn-A1* and *Vrn-D1* increase spring freezing susceptibility, while *Vrn-B1* enhances freezing tolerance [27]. Although primarily involved in flowering regulation, *Vrn-B3* also participates in freezing tolerance-related signaling pathways. These studies have revealed complex relationships between *VRN* loci and freezing tolerance in wheat. However, there is still a knowledge gap in understanding the relationship between the combination of vernalization gene alleles rather than single loci and the multi-stage freezing tolerance of wheat.

*VRN1* and *VRN-B3* genes have been cloned, and molecular markers developed from them [28,29] are widely used in breeding [30,31,32]. In this study, we used 575 wheat accessions to identify the allelic status of genes *Vrn-A1*, *Vrn-B1*, *Vrn-D1,* and *Vrn-B3*, investigate the freezing tolerance at the seedling stage using field tests, and at the jointing stage in climate chamber tests, and analyze the effects of various alleles on freezing tolerance across different growth stages. The results were expected to identify freezing-tolerant germplasm and molecular markers for breeding.

## 2. Results

### 2.1. Analysis of Freezing Tolerance Traits at the Seedling Stage and at the Jointing Stage

**Figure 1:**
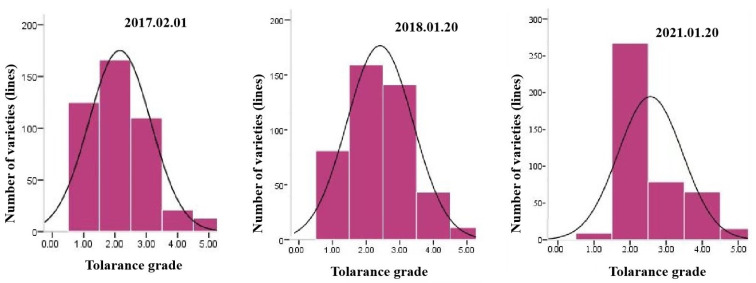
Distributions of freezing tolerance of 435 wheat accessions in three environments.

The accession panel (Table S1) showed considerable variation in freezing tolerance across environments (Table 1). The freezing tolerance grades ranged from one to five, with standard deviations between 0.89 and 0.99, and coefficients of variation (CV) ranging from 34.78% to 46.04% (Table 1). These findings indicated that the three experimental environments effectively differentiated freezing tolerance among genotypes. Histograms showed that the phenotypic distributions for the three environments were normally distributed (Figure 1). The majority of accessions exhibited freezing tolerance grades of one to three across all three environments, indicating a higher proportion of accessions with strong to moderate freezing tolerance. Highly significant correlations were observed among freezing tolerance phenotypes across three environments, with correlation coefficients ranging from 0.699 to 0.773 (Table 2), which demonstrated that the phenotypes were stable across environments.

A total of 435 wheat accessions were collected from four ecological wheat production zones in China: the Northern Winter Wheat Region (NWWR), the Yellow and Huai Valley Winter Wheat Region (YHVWWR), the Middle and Lower Reaches of the Yangtze River Winter Wheat Region (MLRYRWWR), and the Southwest Winter Wheat Region (SWWR). Significant regional variations in freezing tolerance were observed among these accessions. The NWWR accessions (n = 13) exhibited the highest freezing tolerance with an average damage level of 1.7, followed by YHVWWR accessions (n = 268) at 2.0. In contrast, MLRYRWWR (n = 128) and SWWR (n = 26) accessions showed comparatively lower freezing tolerance, with average damage levels of 3.0 and 3.3, respectively. Statistical analysis revealed that the freezing damage grades of NWWR and YHVWWR accessions were significantly lower than those of MLRYRWWR and SWWR accessions (*p* < 0.05; Table S2), clearly demonstrating superior freezing tolerance in wheat varieties from the northern regions (NWWR and YHVWWR) compared to their southern counterparts (MLRYRWWR and SWWR).

**Figure 2:**
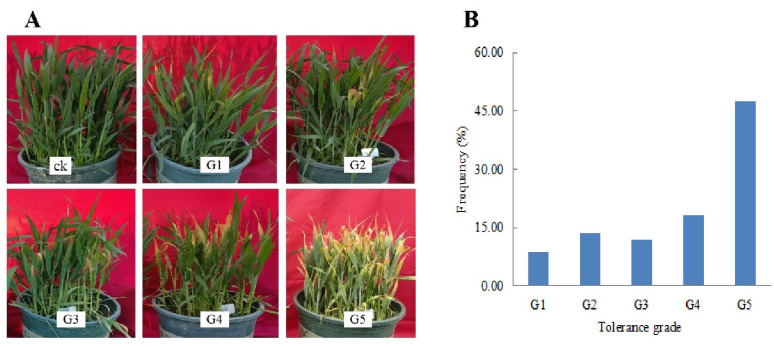
The freezing tolerance of wheat accessions at the jointing stage. (**A**) Growth performance of different tolerance grades of wheat accessions. (**B**) Frequency of freezing tolerance of 192 wheat accessions. Freezing tolerance was rated on a one to five grade: G1 = the strongest tolerance, G5 = the weakest tolerance.

The dead stem rates of 192 accessions showed significant variation, ranging from 0.01 to 1.00 (Table S3) with a mean of 0.16 and a coefficient of variation of 52.24%. The −6 °C/6 h treatment at the jointing stage effectively distinguished differences in freezing tolerance among accessions at this growth stage. Following the dead stem grading standards from Liu et al. [33], freezing tolerance was divided into five tolerance levels (Figure 2A) with the majority in level five (Figure 2B). There were 17 accessions with grade one, and the average dead stem rate was 0.08. There were 26 accessions with grade two, and the average dead stem rate was 0.22. There were 23 accessions with grade three, and the average dead stem rate was 0.37. There were 35 accessions with grade four, and the average dead stem rate was 0.54. There were 91 accessions with grade five, and the average dead stem rate was 0.90 (Table S3). In summary, the freezing tolerance of 192 wheat accessions is obviously different, and the proportion of extremely weak accessions is the largest.

The relationship between seedling-stage and jointing-stage freezing tolerance of 52 accessions grown in the Yellow and Huai Valley Wheat Region is shown in Table 3. Some accessions such as Handan 6172, Huaimai 22, Jimai 22, Yannong 21 and Huaimai 29 had strong freezing tolerance at both growth stages. Accessions such as Annong 1124, Chuanmai 42 and Nemai 8 had strong freezing tolerance at jointing, but weak seedling freezing tolerance in winter; accessions Liangxing 99, Jinan 17 and Guomai 8 had strong seedling freezing tolerance, but weak tolerance in spring. Correlation analysis indicated that freezing tolerance at the two growth stages was not significant.

### 2.2. Association of Seedling Freezing and Vernalization Genotype

**Figure 3:**
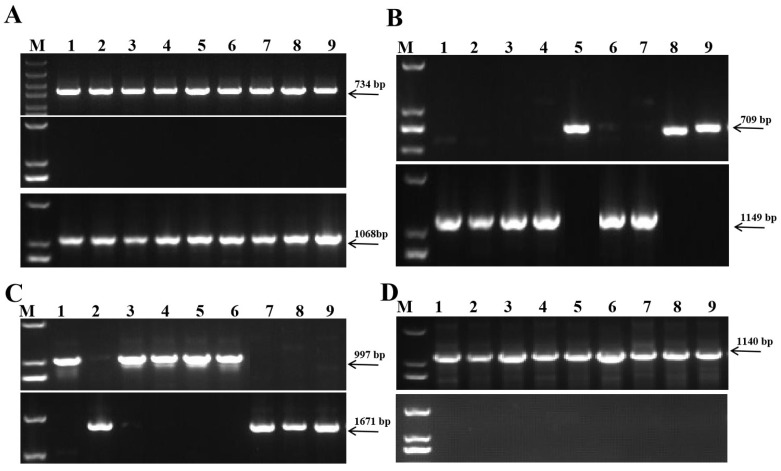
Allelic variation detected in the *VRN-A1*, *VRN-B1*, *VRN-D1* and *VRN-B3* loci among nine wheat accessions. Amplification with (**A**): primers Vrn1-AF/Int1R (uppermost), Intr1-AF2/AR3 (middle) and Intr1-CF/ABR (lowermost); (**B**): primers of Intr-BF/BR3 and Intr-BF/BR4; (**C**): primers Intr1-DF/DR3 and Intr1-DF/DR4; (**D**): primers of VRN4-B-INS-F/R and VRN4-B-NOINS-F/R. M, DL2000; 1, Yannong 19; 2, Yangmai 158; 3, Huaimai 20; 4, Su 553; 5, Huaimai30; 6, Bainong207; 7, Annong 1124; 8, Luo 1106; 9, Qian 110209.

At *VRN-A1* locus, screening of the genotyping of the panel with PCR primer set Vrn1-AF/Int1R indicated that all 435 accessions had the 734-bp fragment for the *vrn-A1* or *Vrn-A1c* alleles. Amplification with primer set Intr1-AF2/AR3 produced no PCR product whereas primer Intr1-CF/ABR produced a fragment of 1068-bp (Figure 3A). Results from the three independent PCRs indicated that all 435 wheat accessions carried the recessive *vrn-A1* allele (Table S1).

Genotyping of the *VRN-B1* locus by PCR primer sets Intr-BF/BR3 and Intr-BF/BR4 (Figure 3B) indicated that 18 accessions had a 709-bp fragment, indicative of the *Vrn-B1* allele, While *vrn-B1* was detected in all other accessions (Table S1).

Genotyping of the *VRN-D1* locus with PCR primer sets VRN4-B-INS-F/R and VRN4-B-NOINS-F/R (Figure 3C) indicated that 178 accessions had the 1671-bp fragment, indicative of *Vrn-D1*, while *vrn-D1* was detected in all other accessions (Table S1).

At *VRN-B3* locus, amplification with primer set VRN4-B-INS-F/R detected no PCR product that identifies *Vrn-B3*; however, all accessions produced a 1140-bp fragment when amplified with primer set VRN4-B-NOINS-F/R (Figure 3D), indicative of *vrn-B3*.

**Figure 4:**
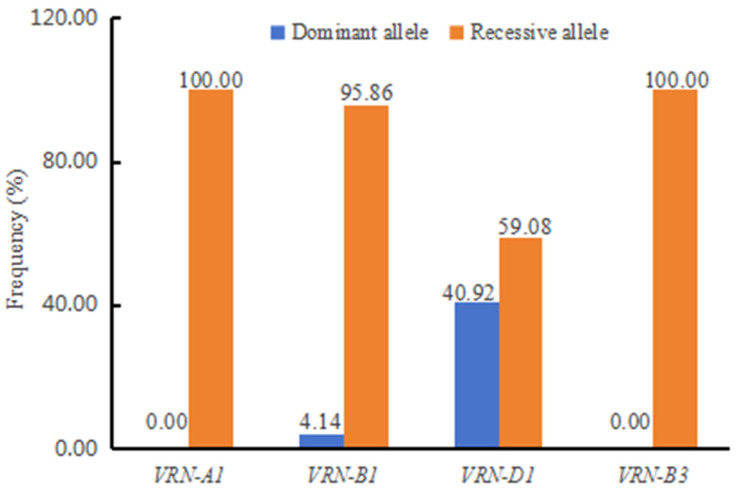
Frequency of alleles of vernalization genes in 435 wheat accessions.

Molecular marker detection results indicated that the highest frequency is the dominant *Vrn-D1* allele, accounting for 40.92% of the tested accessions, and a higher frequency is the dominant *Vrn-B1* allele, accounting for 4.14% of the tested accessions among 435 wheat accessions. We did not find the dominant *Vrn-A1* allele and dominant *Vrn-B3* allele (Figure 4). Characterization of the allelic combination of vernalization genes at *Vrn-A1*, *Vrn-B1*, *Vrn-D1* and *Vrn-B3* loci revealed that there was a total of four types of allelic variation compositions. Among them, there were 242 accessions that possessed the recessive *vrn-A1*/*vrn-B1*/*vrn-D1*/*vrn-B3* allelic variant combinations (accounting for 55.63%). There were 190 accessions carrying one dominant allelic variation; 175 out of 190 accessions had *vrn-A1*/*vrn-B1*/*Vrn-D1*/*vrn-B3* allelic variant combinations (accounting for 40.23%), while 15 accessions possessed *vrn-A1*/*Vrn-B1*/*vrn-D1*/*vrn-B3* allelic variant combinations (accounting for 3.45%; Table 4). Only 3 accessions possessed two dominant allelic variations, which were *vrn-A1*/*Vrn-B1*/*Vrn-D1*/*vrn-B3* allelic variant combinations (accounting for 0.69%). These suggested that the recessive allelic combination of *vrn-A1*/*vrn-B1*/*vrn-D1*/*vrn-B3* was predominant, but the combination of *vrn-A1*/*vrn-B1*/*Vrn-D1*/*vrn-B3* was prevalent in winter wheat.

**Figure 5:**
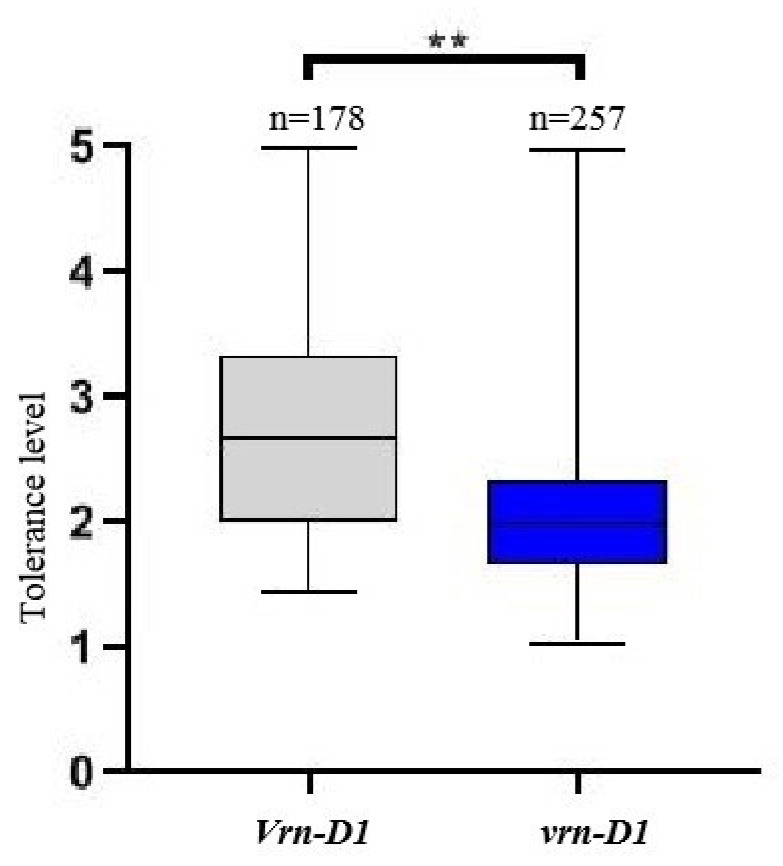
Comparison of freezing tolerance between the *Vrn-D1* and *vrn-D1* genotypes. ** *p* < 0.01.

A significant association was observed between the *Vrn-D1* allele and reduced freezing tolerance in three environments. The *Vrn-D1* allele was positively correlated with freezing tolerance grade with correlation coefficients of 0.288, 0.280, and 0.503, respectively (*p* < 0.01; Table 3). A Mann–Whitney U test also showed that the average freezing tolerance grade of *Vrn-D1* and *vrn-D1* genotypes was significantly different (*p* < 0.01; Figure 5). The *Vrn-B1* allele had no significant correlation with the freezing tolerance grade surveyed in 2017 and 2018, but showed a significant positive correlation with freezing tolerance grade in 2021 although the 0.141 correlation coefficient was very low (Table 3).

The average freezing tolerance level of lines carrying alleles *Vrn-B1* and *Vrn-D1* was 3.00, which was higher than accessions carrying only *Vrn-B1* (2.87) or *Vrn-D1* (2.78). The average freezing damage grade of lines with all four recessive alleles was 2.05 and significantly different from lines with one or two dominant alleles (*p* < 0.05; Table 4). Co-presence of recessive genes at all four loci was prerequisite for strong freezing tolerance in seedling.

### 2.3. Association of Jointing Freezing and Vernalization Genotype

PCR screening of the 192 wheat accessions from the Yellow and Huai Valley Wheat Region using primer set Vrn1-AF/Int1R indicated that all produced the 734-bp fragment, whereas there was no PCR product with primer set Intr1-AF2/AR3and 1068-bp fragment with primer set Intr1-CF/ABR. The combined PCR results indicated that all these accessions had *vrn-A1*. Screening with PCR primer sets Intr-BF/BR3 and Intr-BF/BR4 indicated that three wheat accessions (Bainong 3217, Huaimai 30, and Shan7859) had *Vrn-B1* allele characterized by a 709-bp fragment with primer Intr-BF/BR3; the remaining 189 accessions had *vrn-B1*. Seventy one accessions (59%) harbored *Vrn-D1* allele, and 121 carried *vrn-D1*. PCR results showed that all 192 accessions carried *vrn-B3*. The average dead-stem score of accessions carrying *Vrn-D1* was 0.62; the average score for *vrn-D1* accessions was 0.61, indicating that the *VRN-D1* locus had no obvious relationship with freezing tolerance at the jointing stage.

## 3. Discussion

Freezing stress on seedlings at the beginning of winter and at jointing in spring can injure wheat plants and negatively impact growth, development, and yield. Although the occurrence of frost damage is influenced by various factors, genetic variation among accessions plays a crucial role. In this study, we systematically evaluated the freezing tolerance of wheat at the seedling stage and the jointing stage. Currently, the evaluation of seeding-freezing tolerance of wheat in China follows the industry standard of the People’s Republic (NY/T 1301–2007) [34] which divides freezing symptoms into five grades. Using this assessment method, we phenotyped 435 wheat accessions in three different environments. The highly significant correlations (ranging from 0.699 to 0.773) among the freezing tolerance phenotypes across these environments (Table 2) indicate that the genetic basis for seedling-freezing tolerance is stable and heritable. This stability allows for reliable selection of freezing-tolerant germplasm during breeding.

Yellow and Huai Valley Wheat Region is in the transitional zone between north and south where frequent non-anticipated temperature fluctuations in spring can affect the wheat crop. Hence accessions with strong freezing tolerance especially at the jointing stage would be beneficial to production. The jointing-stage freezing tolerance of wheat is influenced by multiple factors, such as the occurrence-period, intensity and duration of low temperature. Because the occurrence period and intensity of low temperature in field are not consistent among years, it is difficult to get reliable and repeatable results. Artificial simulation identification is characterized by a remarkably short cycle and high repeatability, which enables researchers to efficiently obtain consistent and reliable results [35]. In this study, we applied this pot-planting and artificial simulation approach to evaluate the jointing-freezing tolerance of 192 wheat accessions from the region. Using the dead stem rate as an evaluation index, we found significant differences among different wheat accessions. The −6 °C/6 h treatment at the jointing stage effectively distinguished the genetic differences in freezing tolerance, which is consistent with previous studies [33]. However, compared with the seedling stage, a higher proportion (65.63%) of accessions showed weak freezing tolerance at the jointing stage, indicating that breeding for jointing-stage freezing tolerance is more challenging.

Assessing the freezing tolerance of 52 wheat accessions at both the seeding and jointing stages revealed no significant correlation between tolerance at these two stages, a finding consistent with previous reports by Zhong et al. [36]. This suggests that there may be different genetic mechanisms for the regulation of freezing tolerance at these two stages in wheat. In this study, only 12.19% of accessions showed a lack of seedling freezing tolerance, whereas 65.63% lacked tolerance at jointing. These findings demonstrate that freezing tolerance at the seedling stage is more amenable to selection during breeding compared to that at the jointing stage. Breeding for jointing-freezing tolerance is more challenging and future breeding programs should prioritize enhancing spring freezing tolerance, particularly during critical developmental phases such as the jointing stage.

The distribution frequencies of vernalization gene dominant alleles vary among different regions. In our study, among 435 winter wheat samples, the dominant allele frequencies were *Vrn-D1* (40.92%) > *Vrn-B1* (4.14%), with no detection of *Vrn-A1* or *Vrn-B3*. In the 192 accessions from the Yellow and Huai Valley Wheat Region, *Vrn-D1* was even more predominant at 58.68%, followed by *Vrn-B1* at 1.56%. Comparative analysis with previous studies showed notable differences: Zhang et al. [37] reported higher detection rates for *Vrn-B1* (18.2%) and *Vrn-A1* (13.8%) in broader geographical samples, though *Vrn-D1* (45.3%) remained predominant. Jiang et al. [38] documented a *Vrn-D1* frequency (56.12%) closely aligned with our Yellow and Huai Valley data. These discrepancies primarily stem from sample heterogeneity—prior studies included accessions from northern spring wheat regions, whereas our investigation strictly focused on winter wheat germplasm. By integrating geographical distribution patterns [39], we discerned that the high-frequency occurrence of *Vrn-A1*/*B1* in spring wheat regions directly explains their absence in our winter wheat samples. This robustly confirms that winter wheat accessions are predominantly characterized by the *Vrn-D1* dominant allele, with *Vrn-B1* as a secondary component-a distribution pattern consistent with China’s wheat ecoregionalization mechanisms.

We also analyzed the relationship between vernalization gene alleles and freezing tolerance. At the seedling stage, we found that the dominant gene *Vrn-D1* was positively correlated with the freezing damage grade (Table 3), negatively regulating the freezing tolerance of wheat. The result is strongly associated with Zhang et al. [40], who found the recessive *vrn-D1* allele was more effective than dominant *Vrn-D1* allele in improving winter tolerance of wheat. The average freezing tolerance level of accessions carrying *Vrn-B1* and *Vrn-D1* was higher than that of accessions carrying only one of these dominant alleles. Moreover, the average freezing damage grade of accessions with all four recessive alleles (*vrn-A1*/*vrn-B1*/*vrn-D1*/*vrn-B3*) was significantly lower (2.05) compared to accessions with one or two dominant alleles (Table 4). This indicates that the recessive *VRN1* allelic combination is closely related to strong seedling-freezing tolerance, which is correlated with You et al. [41]. Therefore, under the premise that *VRN-A1* site is recessive, *VRN-D1* gene markers can be effectively used for screening strong seedling-freezing tolerance wheat materials.

However, at the jointing stage, there was no obvious relationship between the vernalization gene and spring freezing tolerance. Although previous studies have shown that some *VRN1* alleles can affect spring freezing tolerance [27], in our study, due to the limited sample size and type, no dominant alleles *Vrn-A1* and *Vrn-B3* were detected in 192 accessions from the Yellow and Huai Valley Wheat Region, and only two samples contained the dominant *Vrn-B1* gene. Despite the high proportion (58.68%) of dominant *Vrn-D1*, there was no difference in the dead stem rate between the recessive *vrn-D1* and dominant *Vrn-D1* accessions. This suggests that other genetic or environmental factors may play more important roles in regulating jointing-freezing tolerance.

In this study, freezing tolerance of 435 wheat germplasm at the seedling stage and 192 wheat germplasm at the jointing stage were systematically evaluated, and allelic variation analysis of *VRN* gene was combined to screen out germplasm resources with significant frost resistance potential. Among the 435 accessions evaluated at the seedling stage, 47 exhibited consistently high freezing tolerance (grade one) in at least two independent environments. Notably, seven accessions—Handan 6172, ENESCO, Shijiazhuang 8, Shijiazhuang 15, Tai 10604, Niavt14, and Gushenmai 9—maintained grade one tolerance across all three testing environments. These accessions carried the recessive allelic combination of *vrn-A1*/*vrn-B1*/*vrn-D1*/*vrn-B3*. Such accessions can serve as valuable genetic resources for breeding wheat varieties with enhanced seedling freezing tolerance. For the 192 wheat accessions from the Yellow and Huai Valley Wheat Region evaluated at the jointing stage, although most of the accessions (65.63%) showed very weak tolerance (Figure 2B), some accessions such as Anke 20, Fengde Cunmai 5, Gu Shen 6, Huacheng 2019, Anke 237, Fengde Cunmai 1, An 1302, Anke 238, Henong 825, Hengjinmai 8, Anke 2101, Bifeng 1, Handan 6172, Huacheng 3366, Anke 239, Huaimai 22, and Xiaoyan 6 showed relatively strong freezing tolerances at the jointing stage. Although the relationship between vernalization genes and jointing-freezing tolerance was not clear in this study, these accessions can still be considered as potential germplasm for improving spring freezing tolerance in wheat breeding programs. Further research on their genetic mechanisms of freezing tolerance may provide new insights into enhancing wheat’s resistance to spring cold stress.

## 4. Materials and Methods

### 4.1. Plant Materials

A total of 435 wheat accessions from different ecological wheat production zones were used for the evaluation of freezing tolerance in seedlings (Table S1). Among them, 268 accessions are from the Yellow and Huai Valley Winter Wheat Region (YHVWWR), 128 are from the Middle and Lower Reaches of the Yangtze River Winter Wheat Region (MLRYRWWR), 26 are from the Southwestern Winter Wheat Region (SWWWR), 13 are from the Northern Winter Wheat Region (NWWR). Since wheat in the Yellow and Huai Valley Wheat Region is frequently affected by freezing injury in spring, we also assessed 192 accessions (52 from the above accessions) from the Yellow and Huai Valley Wheat Region for freezing tolerance at jointing (Table S3).

### 4.2. Assessing of Freezing Tolerance Traits in Seedlings

A total of 435 wheat accessions were grown in field trial at the Huaibei Experiment Station (116°45′ N, 33°54′ E) of Anhui Academy of Agricultural Sciences (Hefei in Anhui province). They were planted in the conventional autumn season on 26 October 2016, 2017 and 2020, which is during the recommended seeding period for winter wheat in this region. The experimental design was a randomized complete block with two replicates. Plot size was 2-m-long rows with row spacing of 25 cm. All trials were seeded by manual dibbling at a seedling rate of 80 seeds row^−1^. The total amount of urea applied during the whole growth period of wheat is 390 kg/hm. The application ratio of the base fertilizer to the top dressing is 7:3. Nitrogen fertilizer should be top-dressed during the jointing stage of wheat, and this should be carried out in combination with irrigation.

Meteorological information was provided by the Weather Bureau of Suixi County, Huaibei City. Frost damaging events occurred during 22~25 January 2017 (environment E1), 19~21 January 2018 (environment E2), and 6~9 January 2021 (environment E3). The lowest temperatures were −8 °C, −12 °C, and −12 °C, respectively. Before freezing stress, the diurnal temperature variation was relatively stable, which is generally suitable for the initial growth and development of wheat. Adequate sunlight ensured normal photosynthesis and promoted the growth of seedlings. The soil moisture content was maintained at a relatively optimal level, which provided sufficient water for the plants. Freezing tolerance phenotypes were recorded two weeks after freezing. According to the agricultural industry standard of China (NY/T1301–2007) [31], freezing tolerance was recorded on a five-grade, as follows:

Grade 1 (G1): no freezing damage;

Grade 2 (G2): leaf yellowing;

Grade 3 (G3): 50% leaf death;

Grade 4 (G4): All leaves dead or withered;

Grade 5 (G5): entire plants or most tillers dead.

### 4.3. Assessing Freezing Tolerance Traits in Spring

This experiment was conducted in 2020–2021 at the experimental station of Anhui Academy of Agricultural Sciences (31.83° N, 117.24° E), Hefei, China. The 192 wheat accessions were used in the experimentation. Wheat accessions were sown in pots of 28 cm diameter × 35 cm height, on 4 November 2021. There were 13 pots for each accession. Each pot was filled with 8 kg soil and 5.00 g compound fertilizer (N:P:K = 15:15:15) incorporated in it. The soil was taken from the field of 0∼20 cm upper plowing layer. Twenty seeds were planted in each pot and seedlings were thinned to ten at the three-leaf stage. All the pots were placed in the field conditions. The field environment for wheat growth is suitable, with stable day-night temperatures, ample sunlight, and appropriate soil moisture, creating favorable conditions for the growth of wheat.

When the plants reached the jointing stage (ACFP), eight pots of uniformly grown wheat plants from each accession were moved to a climate chamber for exposure to −6 °C for 6 h (humidity: 60%; light intensity: 0 µmol m^−2^ s^−1^). The pots were moved back to the field after the treatment. The numbers of dead main stem and first and second tillers were assessed after 10 days of low temperature treatment. The dead-stem rate per pot was calculated as: Dead-stem rate = number of dead stems/total number of stems. The mean dead-stem rate was calculated for each accession using 8 replicated pots, and the tolerance grade of wheat accessions were classified based on these mean values. According to the criteria of jointing-freezing tolerance [30], the jointing-freezing tolerance of wheat was divided into 5 grades (Table 5).

### 4.4. Molecular Marker Detection

Genomic DNA (gDNA) was extracted from young leaves of ten-day-old seedlings using the phenol chloroform method [42]. DNA concentration and quality were checked with NanoDrop2000 (Thermo Scientific, Waltham, MA, USA). DNA samples with a 260 nm/280 nm ratio equal to or higher than 1.8 were considered suitable for further PCR analysis. Nine functional markers (Table S4) specific for *Vrn-A1*, *Vrn-B1*, *Vrn-D1* and *Vrn-B3* alleles [10,26,27] were used to genotype all accessions. Primers were synthesized by Sangon Biological Engineering Technology and Service Co., Ltd. (Shanghai, China). DNA amplification was carried out in 20-µL reaction volumes, each consisting of 1 µL of 50–100 ng/µL DNA, 1 µL of 10 µmol/L of each primer, 10 µL of 2 × Taq PCR Master Mix (Tsingke Biotechnology Co., Ltd., Beijing, China), and 7 µL of sterilized ddH_2_O. The annealing temperature and extension time used for the PCR are provided in Supplementary Materials Table S4. PCR products were separated in 1–3% agarose gels depending on the PCR product size (Table S4) and visualized under UV light after staining with ethidium bromide.

### 4.5. Statistical Analysis

Phenotypic differences in freezing tolerance among accessions were tested using analysis of variance (ANOVA) in the SPSS software 20.0, and multiple comparisons were made using the least significant difference (LSD) test at *p* < 0.05.

## 5. Conclusions

*Vernalization* genes play an important role in seedling-stage freezing tolerance of wheat. The *VRN-D1* molecular marker can be used as an effective tool for screening freezing-tolerant accessions at the seedling stage. However, jointing-stage freezing tolerance did not show a significant association with *VRN* genotypes, which may involve other low-temperature responsive genes or interactions with environmental factors. Han 6172, Huai Mai 29, and other germplasms with strong freezing tolerance at both the seedling and jointing stages were selected, which provided the core parents for multi-stage resistance breeding. Future studies should further analyze the molecular basis of freezing tolerance at the jointing stage, develop efficient molecular markers, and integrate phenomics with gene-editing technologies to accelerate the cultivation of new wheat varieties exhibiting broad adaptability to climate change.

## Figures and Tables

**Table 1:** Phenotypic variation of freezing tolerance of 435 wheat accessions.

Years	Minimum Temperature (°C)	Grade Range	Mean	SD	CV (%)
2017	−8	1–5	2.15	0.99	46.04
2018	−12	1–5	2.41	0.98	40.76
2021	−12	1–5	12.56	0.89	34.78

SD: Standard deviation, CV: Coefficient of variation, Freezing tolerance was rated on a one to five scale: one = the strongest tolerance, five = the weakest tolerance.

**Table 2:** Correlations of phenotypic data and genotypes of 435 wheat accessions in different environments.

	Freezing Tolerance			Gene Type	
	2017	2018	2021	*Vrn-D1*	*Vrn-B1*
2017	1				
2018	0.773 **	1			
2021	0.699 **	0.702 **	1		
*Vrn-D1*	0.288 **	0.280 **	0.503 **	1	—
*Vrn-B1*	0.050	0.078	0.141 **	—	1

** *p* < 0.01; — Not determined.

**Table 3:** The freezing grade of 52 winter wheat accessions.

Accession	Seedling-Freezing Grade (In the Field)			Jointing-Freezing Grade (In the Climate Chamber)
	2017	2018	2021	
Fengdecunmai 5	3	2	2	1
Henong 825	2	2	2	1
Handan 6172	1	1	1	1
Huaimai 22	1	1	2	1
Xiaoyan 6	2	1	2	1
Annong1124	3	3	4	2
Bainon g3217	2	2	2	2
Chuanmai 42	3	4	4	2
Jimai 22	1	1	2	2
Huaimai 30	2	3	3	2
Huaimai 29	1	1	2	2
Huiyan 22	1	2	3	2
Neimai 8	5	4	4	2
Yannong 21	1	1	2	2
An1243	1	2	2	2
Bainong 207	3	3	2	2
Huaimai 18	1	1	2	3
Huaimai 20	1	1	2	3
Luyuan 502	2	3	3	3
Yannong 19	1	1	2	3
Huaimai 25	1	1	3	3
Shijiazhuang 8	1	1	1	3
Yangnuomai 1	5	5	5	3
Zhengmai 366	2	2	2	3
Su 553	1	2	2	4
Aikang 58	1	2	2	4
Fanmai 5	2	2	3	4
Yanzhan 4110	2	2	3	4
Jimai 73	1	1	2	4
Zhengmai 9023	1	2	2	4
Wanmai 38	1	1	2	4
Zhoumai 18	2	3	2	4
Yannong 999	2	2	2	4
Jinan 17	1	1	2	4
Zhongmai 895	3	2	2	4
Luanxuan 988	3	2	2	5
Xinong 889	2	2	2	5
Qianmai 18	3	3	2	5
Guomai 8	1	1	2	5
Huiyan 77	1	2	3	5
Xinmai 18	2	3	2	5
Jimai 20	2	2	2	5
Kaimai 18	3	3	2	5
Yangmai 20	3	4	3	5
Liangxing 99	1	1	2	5
Mianmai 39	3	2	3	5
Guoshengmai 1	3	4	4	5
Yangmai 158	4	3	4	5
Wanmai 52	1	2	2	5
Ligao 6	1	2	2	5
Guinong 775	2	2	1	5
Neimai 836	5	4	4	5

**Table 4:** Effects of vernalization genotype on freezing grade.

Gene Type	Freezing Grade (Mean ± SD)	Number of Accessions	Frequency (%)
*vrn-A1* + *vrn-B1* + *vrn-D1* + *vrn-B3*	2.05 ± 0.69 a ^1^	242	55.63
*vrn-A1* + *vrn-B1* + *Vrn-D1* + *vrn-B3*	2.78 ± 0.90 b	175	40.23
*vrn-A1* + *Vrn-B1* + *vrn-D1* + *vrn-B3*	2.87 ± 0.77 b	15	3.45
*vrn-A1* + *Vrn-B1* + *Vrn-D1* + *vrn-B3*	3.00 ± 0.33 b	3	0.69

^1^ Different lowercase letters indicate significant differences at *p* < 0.05.

**Table 5:** Evaluation criteria of jointing-freezing tolerance.

Tolerance Grade	Dead Stem Rate	Tolerance Type
1	0.00~0.13	Extremely strong
2	0.14~0.28	Strong
3	0.29~0.42	Moderate
4	0.43~0.65	Weak
5	0.66~1.00	Extremely weak

## Data Availability

Data are contained within the article and Supplementary Materials.

## Supplementary Materials

The following supporting information can be downloaded at: https://www.mdpi.com/article/10.3390/plants14091350/s1. Table S1. The Seedling-freezing grade and vernalizing genotypes of 435 wheat accessions. Table S2. The differences in freezing tolerance across different ecological regions. Table S3. The jointing-freezing tolerance and vernalizing genotypes of 192 wheat varieties. Table S4. Primer sequences used to identify *VRN-1* and *VRN-B3* alleles.
