# Supplementary material for: Winter Wheat Vernalization Alleles and Freezing Tolerance at the Seedling and Jointing Stages
Source: Plants (Basel). 2025 Apr 30;14(9):1350. doi: 10.3390/plants14091350 (PMC12073134; doi:10.3390/plants14091350)
Supplement: Supplementary file 1 [file plants-14-01350-s001.zip › Supplementary material.pdf]

Table S1 The Seedling-freezing grade and vernalizing genotypes of 435 wheat accessions

**No**	**Accessions**	**Ecological region**	Seedling-freezing grade			*VRN-A1*	*VRN-B1*	*VRN-D1*	*VRN-B3*
			**2017**	**2018**	**2021**				
1	Yangmai 158	MLRYRWWR	4	3	4	R	R	D	R
2	876	MLRYRWR	1	2	3	R	R	R	R
3	9987	MLRYRWR	2	3	2	R	R	D	R
4	984121	YHVWWR	2	2	2	R	R	R	R
5	02-192	MLRYRWWR	3	4	3	R	R	R	R
6	02P67	MLRYRWWR	3	3	4	R	R	R	R
7	02Y151	MLRYRWWR	3	4	4	R	R	R	R
8	03-885	MLRYRWWR	4	3	4	R	R	D	R
9	03G7	YHVWWR	1	1	2	R	R	R	R
10	11-6005	MLRYRWWR	3	3	3	R	D	D	R
11	11-6009	MLRYRWWR	3	4	4	R	R	D	R
12	AR2	MLRYRWWR	4	4	5	R	R	R	R
13	ARZ	YHVWWR	4	4	5	R	R	R	R
14	B41011-1-8-4	MLRYRWWR	2	3	2	R	R	R	R
15	B80205-1-2	MLRYRWWR	2	3	2	R	R	R	R
16	B80488-1-1	YHVWWR	2	3	2	R	D	R	R
17	CP01-27-3-1-1	YHVWWR	2	2	3	R	R	D	R
18	CP01-39-17-1	YHVWWR	2	3	2	R	R	R	R
19	CP01-39-3-2-4	YHVWWR	1	1	2	R	R	R	R
20	CP02-62-1-2-2-3	YHVWWR	1	2	2	R	R	R	R
21	CP02-63-13-1	YHVWWR	1	2	2	R	R	D	R
22	CP02-8-5-6-1	YHVWWR	2	1	1	R	R	R	R
23	CP02-8-5-6-2	YHVWWR	2	2	3	R	R	D	R
24	CP02-9-3-1-1-1	YHVWWR	1	1	2	R	R	D	R
25	CP06-11-10-1	YHVWWR	1	1	2	R	R	R	R
26	CP06-31-7-1-1	YHVWWR	1	1	2	R	R	R	R
27	CP06-69-3-2-1	YHVWWR	1	2	2	R	R	R	R
28	CP20-30-1-5-2-2-2-1-1	YHVWWR	1	1	2	R	R	R	R
29	CP20-35-1-5-1-1-1	YHVWWR	1	1	2	R	R	R	R
30	CP20-39-11-1	YHVWWR	2	2	3	R	R	D	R
31	DH6197	MLRYRWWR	3	4	4	R	R	D	R
32	E158	MLRYRWWR	2	2	2	R	R	R	R
33	ENESCO	YHVWWR	1	1	1	R	R	R	R
34	FARO	MLRYRWWR	3	3	3	R	R	D	R
35	Glenlen	MLRYRWWR	5	5	5	R	D	R	R
36	GR123	YHVWWR	2	3	2	R	R	R	R
37	HHW08-01	YHVWWR	1	1	2	R	R	R	R

38	LS6045	YHVWWR	1	1	2	R	R	R	R
39	Niavt14	NWWR	1	1	1	R	R	R	R
40	Nick	MLRYRWWR	5	5	5	R	R	R	R
41	R146	MLRYRWWR	2	3	2	R	R	D	R
42	R77	MLRYRWWR	3	3	4	R	R	R	R
43	RL6077	MLRYRWWR	5	5	5	R	R	D	R
44	TQ0908	YHVWWR	2	2	2	R	R	R	R
45	TQ1203	YHVWWR	1	2	2	R	R	D	R
46	TupatecoR	MLRYRWWR	3	4	4	R	R	D	R
47	W1028	YHVWWR	2	3	2	R	R	R	R
48	X9610	MLRYRWWR	2	3	3	R	R	D	R
49	Y14	MLRYRWWR	3	3	3	R	R	D	R
50	Y18	MLRYRWWR	3	3	3	R	R	D	R
51	Aifengzao 8	YHVWWR	2	3	3	R	R	D	R
52	Aikang 58	YHVWWR	1	2	2	R	R	R	R
53	Aizao 64xi	MLRYRWWR	2	2	3	R	R	D	R
54	Annong 0817	YHVWWR	2	2	2	R	R	D	R
55	Annong 1211	YHVWWR	1	3	2	R	R	R	R
56	Annong 1243	YHVWWR	1	2	2	R	R	R	R
57	Annong 0807	MLRYRWWR	1	2	2	R	R	R	R
58	Annong 0822	MLRYRWWR	2	2	2	R	R	R	R
59	Annong 0928	MLRYRWWR	2	3	2	R	R	R	R
60	Annong 0932	MLRYRWWR	3	3	3	R	R	D	R
61	Annong 0942	MLRYRWWR	3	4	4	R	R	D	R
62	Annong 0942-13	MLRYRWWR	4	4	5	R	R	D	R
63	Annong 0950	MLRYRWWR	3	2	3	R	R	D	R
64	Annong 1001	MLRYRWWR	3	2	3	R	R	D	R
65	Annong 1003	MLRYRWWR	2	1	2	R	R	R	R
66	Annong 1004	MLRYRWWR	2	1	2	R	R	R	R
67	Annong 1005	MLRYRWWR	2	1	2	R	R	R	R
68	Annong 1006	MLRYRWWR	2	2	2	R	R	R	R
69	Annong 1007	MLRYRWWR	1	2	3	R	R	D	R
70	Annong 1008	MLRYRWWR	2	2	2	R	R	R	R
71	Annong 1014	MLRYRWWR	2	3	2	R	R	R	R
72	Annong 1016	MLRYRWWR	2	2	2	R	R	R	R
73	Annong 1020	MLRYRWWR	2	2	2	R	R	R	R
74	Annong 1025	MLRYRWWR	1	1	2	R	R	R	R
75	Annong 1026	MLRYRWWR	2	2	2	R	R	R	R
76	Annong 1027	MLRYRWWR	2	2	2	R	R	R	R
77	Annong 1034	MLRYRWWR	3	3	4	R	R	D	R
78	Annong 1039	MLRYRWWR	3	3	4	R	R	D	R

79	Annong 1101	MLRYRWWR	2	3	2	R	R	R	R
80	Annong 1102	MLRYRWWR	2	3	2	R	R	R	R
81	Annong 1103	MLRYRWWR	2	2	2	R	R	R	R
82	Annong 1104	MLRYRWWR	2	2	2	R	R	R	R
83	Annong 1105	MLRYRWWR	1	2	2	R	R	R	R
84	Annong 1106	MLRYRWWR	1	2	2	R	R	R	R
85	Annong 1107	MLRYRWWR	1	2	2	R	R	R	R
86	Annong 1108	MLRYRWWR	2	1	2	R	R	R	R
87	Annong 1109	MLRYRWWR	2	2	2	R	R	R	R
88	Annong 1110	MLRYRWWR	2	3	2	R	R	R	R
89	Annong 1111	MLRYRWWR	3	2	2	R	R	R	R
90	Annong 1112	MLRYRWWR	5	5	5	R	R	D	R
91	Annong 1113	MLRYRWWR	5	5	5	R	R	D	R
92	Annong 1114	MLRYRWWR	3	3	4	R	R	D	R
93	Annong 1115	MLRYRWWR	5	5	4	R	R	D	R
94	Annong 1116	MLRYRWWR	4	5	4	R	R	D	R
95	Annong 1117	MLRYRWWR	3	3	4	R	R	D	R
96	Annong 1118	MLRYRWWR	4	3	4	R	R	D	R
97	Annong 1119	MLRYRWWR	3	4	5	R	R	D	R
98	Annong 1120	MLRYRWWR	3	3	4	R	R	D	R
99	Annong 1121	MLRYRWWR	3	3	4	R	R	D	R
100	Annong 1122	MLRYRWWR	3	3	4	R	R	D	R
101	Annong 1123	MLRYRWWR	3	3	4	R	R	D	R
102	Annong 1124	MLRYRWWR	3	3	4	R	R	D	R
103	Annong 1125	MLRYRWWR	3	3	2	R	R	D	R
104	Annong 1126	MLRYRWWR	4	4	4	R	R	D	R
105	Annong 1127	MLRYRWWR	3	4	4	R	R	D	R
106	Annong 1128	MLRYRWWR	3	3	4	R	R	D	R
107	Annong 1129	MLRYRWWR	3	4	4	R	R	D	R
108	Annong 1130	MLRYRWWR	3	4	4	R	R	R	R
109	Annong 1131	MLRYRWWR	3	3	4	R	R	D	R
110	Annong 1206	MLRYRWWR	2	2	2	R	R	R	R
111	Annong 8455	MLRYRWWR	2	2	2	R	R	D	R
112	Annong 9248W	MLRYRWWR	2	3	3	R	D	R	R
113	Annong 9267	MLRYRWWR	1	2	2	R	R	R	R
114	Annongda 2012-6	MLRYRWWR	1	1	2	R	R	R	R
115	Bainong 207	YHVWWR	3	3	2	R	R	R	R
116	Bainong 3271	YHVWWR	2	2	2	R	R	R	R
117	Bainong 64	YHVWWR	2	2	2	R	R	R	R
118	Bainong 898	YHVWWR	2	1	2	R	R	R	R
119	Bainongaikang 58	YHVWWR	2	2	2	R	R	D	R

120	Baifeng 10-82	YHVWWR	1	2	2	R	R	R	R
121	Baifeng 09-82	YHVWWR	3	2	2	R	R	R	R
122	Bolunxuan 182	YHVWWR	3	3	2	R	R	R	R
123	Caizhi 24	YHVWWR	2	2	2	R	R	R	R
124	Chuanmai 42	SWWWR	3	4	4	R	R	D	R
125	Chuanmai 42 (bai)	SWWWR	3	4	4	R	R	D	R
126	Chuanmai 42 (hong)	SWWWR	4	3	4	R	R	D	R
127	Cunmai 8	YHVWWR	3	3	2	R	R	R	R
128	Danshi 802	YHVWWR	1	2	2	R	R	R	R
129	Danshi 819	YHVWWR	2	3	2	R	R	R	R
130	Dangmai 2	MLRYRWWR	3	3	4	R	R	D	R
131	Dehongfumai 2	YHVWWR	2	2	2	R	R	R	R
132	Dehongfumai 6	YHVWWR	3	3	3	R	R	R	R
133	Dinghong 1208	MLRYRWWR	2	3	3	R	R	R	R
134	Fanmai 12	YHVWWR	3	3	3	R	R	R	R
135	Fanmai 14	YHVWWR	3	3	3	R	R	D	R
136	Fanmai 5039	YHVWWR	2	2	2	R	R	R	R
137	Fanmai 5	YHVWWR	2	2	3	R	R	D	R
138	Fanmai 8	YHVWWR	3	3	2	R	R	R	R
139	Fengdecunmai 12	YHVWWR	2	3	2	R	R	R	R
140	Fengdecunmai 5	YHVWWR	3	2	2	R	R	R	R
141	Fenghua 8829	YHVWWR	1	2	2	R	R	R	R
142	Fu 0382	YHVWWR	3	2	2	R	R	R	R
143	Gaoyou 2018	YHVWWR	1	1	2	R	R	R	R
144	Gushenmai 9	YHVWWR	1	1	1	R	R	R	R
145	Guanmai 1	YHVWWR	3	2	2	R	R	R	R
146	Guanmai 2	YHVWWR	3	3	2	R	R	R	R
147	Guinong 775	SWWWR	2	2	1	R	R	R	R
148	Guomai 0526	MLRYRWWR	2	2	3	R	R	D	R
149	Guomai 10	YHVWWR	2	1	2	R	R	R	R
150	Guoshengmai 1	YHVWWR	3	4	4	R	R	D	R
151	Guoshengmai 5	YHVWWR	1	2	2	R	R	D	R
152	Handan 6172	YHVWWR	1	1	1	R	R	R	R
153	Haomai 16	MLRYRWWR	2	3	4	R	R	D	R
154	Henong 825	NWWR	2	2	2	R	R	R	R
155	Heng 09-6186	YHVWWR	1	1	2	R	R	R	R
156	Hengguan 35	YHVWWR	2	3	2	R	R	D	R
157	Hongwan 3	MLRYRWWR	1	2	2	R	R	D	R
158	Huapei 8	YHVWWR	3	3	2	R	R	R	R
159	Huacheng 69	YHVWWR	1	1	2	R	R	R	R
160	Huacheng 702	YHVWWR	3	2	2	R	R	R	R

161	Huahui 1088	YHVWWR	2	2	2	R	R	R	R
162	Huarui 0712	YHVWWR	2	1	2	R	R	D	R
163	Huaichuan 919	YHVWWR	2	3	3	R	R	D	R
164	Huaihe 6	YHVWWR	1	1	3	R	R	R	R
165	Huaihe 0308	YHVWWR	2	1	2	R	R	R	R
166	Huaihe 0615	YHVWWR	1	1	2	R	R	R	R
167	Huaihe 09104	YHVWWR	2	2	2	R	R	D	R
168	Huaimai 0208	YHVWWR	1	1	2	R	R	D	R
169	Huaimai 0209	YHVWWR	2	1	2	R	R	D	R
170	Huaimai 0226	YHVWWR	1	1	2	R	R	R	R
171	Huaimai 0320	YHVWWR	2	2	2	R	R	R	R
172	Huaimai 0360	YHVWWR	1	1	2	R	R	R	R
173	Huaimai 05155	YHVWWR	2	2	2	R	D	R	R
174	5Huaimai 0615	YHVWWR	1	2	3	R	R	D	R
175	Huaimai 0705	YHVWWR	1	2	3	R	R	D	R
176	Huaimai 0882	YHVWWR	1	2	2	R	R	R	R
177	Huaimai 8	YHVWWR	1	2	2	R	R	R	R
178	Huaimai 11	YHVWWR	2	2	2	R	R	D	R
179	Huaimai 16	YHVWWR	2	3	3	R	R	R	R
180	Huaimai 17	YHVWWR	1	1	2	R	R	D	R
181	Huaimai 18	YHVWWR	1	1	2	R	R	D	R
182	Huaimai 20	YHVWWR	1	1	2	R	R	R	R
183	Huaimai 21	YHVWWR	1	2	2	R	R	R	R
184	Huaimai 22	YHVWWR	1	1	2	R	R	D	R
185	Huaimai 23	YHVWWR	1	2	3	R	R	D	R
186	Huaimai 24	YHVWWR	1	1	3	R	R	D	R
187	Huaimai 25	YHVWWR	1	1	3	R	R	D	R
188	Huaimai 26	YHVWWR	1	1	2	R	R	R	R
189	Huaimai 28	YHVWWR	2	2	3	R	R	D	R
190	Huaimai 29	YHVWWR	1	1	2	R	R	R	R
191	Huaimai 30	YHVWWR	2	3	3	R	D	R	R
192	Huaimai 31	YHVWWR	1	1	2	R	R	R	R
193	Huaishi 0806	YHVWWR	2	1	2	R	R	R	R
194	Huaishi 0808	YHVWWR	1	2	2	R	R	R	R
195	Huaishi 0916	YHVWWR	1	2	2	R	R	R	R
196	Huinong 98	YHVWWR	4	3	3	R	R	D	R
197	Huiyan 22	YHVWWR	1	2	3	R	R	D	R
198	Huiyan 77	YHVWWR	1	2	3	R	R	D	R
199	Jimai 19	YHVWWR	2	2	2	R	R	R	R
200	Jimai 20	YHVWWR	2	2	2	R	R	R	R
201	Jimai 21	YHVWWR	2	1	2	R	R	R	R

202	Jimai 22	YHVWWR	1	1	2	R	R	R	R
203	Jinan 17	YHVWWR	1	1	2	R	R	R	R
204	Ji 5265	YHVWWR	2	2	2	R	R	R	R
205	Jimai 325	YHVWWR	1	1	2	R	R	D	R
206	Jishi 02-1	YHVWWR	2	3	2	R	R	R	R
207	Jiabao 1	YHVWWR	1	2	2	R	R	R	R
208	Jianmai 206	YHVWWR	2	2	2	R	R	D	R
209	Jinhe 9123	YHVWWR	1	2	2	R	R	R	R
210	Jinli 106	YHVWWR	3	2	2	R	R	R	R
211	Jinli 88	YHVWWR	3	3	2	R	R	R	R
212	Jinmai 73	NWWR	1	1	2	R	R	R	R
213	Junmai 35	YHVWWR	1	2	2	R	R	R	R
214	Kaimai 18	YHVWWR	3	3	2	R	R	R	R
215	Kelin 0369	YHVWWR	3	3	2	R	R	R	R
216	Lankao 181	YHVWWR	2	3	2	R	R	R	R
217	Lankao 298	YHVWWR	2	1	2	R	R	R	R
218	Lemai 091156	YHVWWR	2	2	2	R	R	D	R
219	Lemai 102070	MLRYRWWR	1	2	2	R	R	R	R
220	Ligao 6	YHVWWR	1	2	2	R	R	R	R
221	Lian 9791	YHVWWR	1	1	2	R	R	D	R
222	Liangxing 66	YHVWWR	1	1	2	R	R	R	R
223	Liangxing 99	YHVWWR	1	1	2	R	R	D	R
224	Linyou 8159	NWWR	1	1	2	R	R	R	R
225	Longke 10135	MLRYRWWR	4	4	4	R	R	D	R
226	Longke 0901	YHVWWR	1	1	2	R	R	R	R
227	Longke 1211	YHVWWR	1	1	2	R	R	R	R
228	Longke 1221	YHVWWR	1	1	2	R	R	D	R
229	Longmai 109	YHVWWR	1	1	2	R	R	R	R
230	Longanmai 968	YHVWWR	1	2	2	R	R	R	R
231	Luyuan 502	YHVWWR	1	2	2	R	R	D	R
232	Lunxuan 21	MLRYRWWR	2	2	3	R	R	D	R
233	Lunxuan 988	YHVWWR	3	2	2	R	R	D	R
234	Luomai 05159	YHVWWR	2	2	2	R	R	R	R
235	Luomai 31	YHVWWR	3	3	2	R	R	R	R
236	Luo 1104	YHVWWR	2	3	2	R	R	D	R
237	Luo 1106	YHVWWR	2	3	2	R	D	D	R
238	Luo 2267	YHVWWR	3	3	4	R	R	D	R
239	Luo 2708	YHVWWR	3	3	2	R	R	R	R
240	Luo 3249	YHVWWR	3	3	2	R	R	R	R
241	Luo 4688	YHVWWR	3	3	2	R	R	R	R
242	Luo 6073	YHVWWR	3	3	2	R	R	R	R

243	Luo 6078	YHVWWR	3	3	4	R	R	D	R
244	Luo 6099	YHVWWR	2	3	3	R	R	D	R
245	Luo 6135	YHVWWR	3	3	3	R	R	D	R
246	Luo 9920	YHVWWR	3	3	2	R	R	D	R
247	Mianmai 37	SWWWR	4	4	4	R	D	R	R
248	Mianmai 39	SWWWR	3	2	3	R	R	D	R
249	Mianmai 40	SWWWR	3	3	4	R	R	D	R
250	Mingtian 0417	YHVWWR	1	2	2	R	R	R	R
251	Mingtian 07112	YHVWWR	2	1	2	R	R	R	R
252	Niemai 10	SWWWR	3	4	4	R	R	R	R
253	Niemai 836	SWWWR	5	4	4	R	R	R	R
254	Niemai 8	SWWWR	5	4	4	R	R	R	R
255	Niemai 9	SWWWR	4	5	5	R	R	R	R
256	Niexiang 203	YHVWWR	2	2	2	R	R	R	R
257	Ning 0717	MLRYRWWR	3	4	4	R	R	D	R
258	Ningqing 185	MLRYRWWR	2	3	4	R	R	D	R
259	Anda 1108	YHVWWR	2	4	2	R	R	D	R
260	Puxing 5	YHVWWR	2	3	2	R	R	R	R
261	Qian 079939-5	SWWWR	3	4	4	R	R	D	R
262	Qian 079984-14	SWWWR	3	3	2	R	R	R	R
263	Qian 090304-4	SWWWR	2	3	2	R	D	R	R
264	Qian1 102032-8	SWWWR	2	3	3	R	R	D	R
265	Qian 102032-8	SWWWR	2	3	2	R	D	R	R
266	Qian 110209-4	SWWWR	3	3	3	R	D	D	R
267	Qian 11240-2	SWWWR	3	3	3	R	D	R	R
268	Qian 11AF19-5	SWWWR	3	3	4	R	R	D	R
269	Qianmai 18	SWWR	3	3	2	R	R	R	R
270	Qianyu 21	SWWWR	3	4	4	R	R	D	R
271	Quanmai 1094	YHVWWR	1	2	2	R	R	R	R
272	Quanmai 890	YHVWWR	2	2	2	R	R	D	R
273	Ruihua 1101	YHVWWR	3	3	3	R	D	R	R
274	Ruiquanmai 168	YHVWWR	2	3	2	R	R	D	R
275	Shannong 05-066	YHVWWR	1	1	2	R	R	R	R
276	Shannong 055843	YHVWWR	3	1	2	R	R	R	R
277	Shannong 10-2	YHVWWR	1	2	2	R	R	R	R
278	Shannong 785	YHVWWR	1	1	2	R	R	D	R
279	Shannong 56	YHVWWR	3	2	2	R	R	R	R
280	Shannong 70	YHVWWR	2	3	2	R	D	R	R
281	Shengxuan 3	MLRYRWWR	3	4	3	R	R	D	R
282	Shijiazhuang B03-5672	YHVWWR	2	2	2	R	R	R	R
283	Shijiazhuang 15	YHVWWR	1	1	1	R	R	R	R

284	Shijiazhuang 8	YHVWWR	1	1	1	R	R	R	R
285	Shimai 12	YHVWWR	2	2	3	R	R	D	R
286	Shimai 18	YHVWWR	2	2	2	R	R	R	R
287	Shiyou 17	YHVWWR	2	2	2	R	R	R	R
288	Sui 1216	YHVWWR	1	1	2	R	R	R	R
289	Taikong 10604	NWWR	1	1	1	R	R	R	R
290	Taikong 6	YHVWWR	3	3	2	R	R	D	R
291	Tai 18	YHVWWR	1	1	2	R	R	D	R
292	Tianbao 518	MLRYRWWR	2	3	2	R	R	R	R
293	Tianmin 198	YHVWWR	3	4	3	R	R	R	R
294	Wanke 06290	MLRYRWWR	2	2	2	R	R	R	R
295	Wanke 08585	MLRYRWWR	2	3	2	R	R	R	R
296	Wanke 09636	MLRYRWWR	3	3	4	R	R	D	R
297	Wanke 700	MLRYRWWR	1	2	2	R	R	R	R
298	Wankenmai 081	MLRYRWWR	2	3	3	R	R	R	R
299	Wanmai 38	YHVWWR	1	1	2	R	R	R	R
300	Wannong 07213	YHVWWR	2	2	3	R	R	D	R
301	Wannong 606	MLRYRWWR	1	1	2	R	R	D	R
302	Weilai 6193	MLRYRWWR	2	2	3	R	R	R	R
303	Guomai 06011	YHVWWR	1	2	2	R	R	R	R
304	Guomai 0608	YHVWWR	1	2	2	R	R	D	R
305	Guomai 0801	YHVWWR	3	3	3	R	R	D	R
306	Guomai 10	YHVWWR	2	1	2	R	R	R	R
307	Guomai 12	YHVWWR	1	2	2	R	R	R	R
308	Guomai 8	YHVWWR	1	1	2	R	R	R	R
309	Xinong 1211	YHVWWR	2	2	2	R	R	R	R
310	Xinong 266	YHVWWR	3	3	2	R	R	R	R
311	Xinong 529	YHVWWR	4	4	4	R	R	D	R
312	Xinong 585	YHVWWR	2	2	3	R	R	R	R
313	Xinong 622	YHVWWR	3	3	2	R	R	R	R
314	Xinong 889	YHVWWR	2	2	2	R	R	R	R
315	Xianmai 2	YHVWWR	3	3	2	R	R	R	R
316	Xianmai 68	MLRYRWWR	3	3	3	R	R	D	R
317	Xiannong 1	YHVWWR	2	3	2	R	R	R	R
318	Xiaoyan 6	YHVWWR	2	1	2	R	R	R	R
319	Xinmai 0208	YHVWWR	1	2	2	R	R	R	R
320	Xinmai 0401	YHVWWR	2	2	2	R	R	D	R
321	Xinmai 18	YHVWWR	2	3	2	R	R	R	R
322	Xinmai 19	YHVWWR	2	3	2	R	R	D	R
323	Xinmai 19023	YHVWWR	2	3	2	R	D	R	R
324	Xinmai 2111	YHVWWR	3	4	4	R	R	D	R

325	Xinmai 2119	YHVWWR	2	2	2	R	R	R	R
326	Xinmai 23	YHVWWR	2	3	3	R	R	R	R
327	Xinmai 31	YHVWWR	2	2	2	R	R	R	R
328	Xinmai 9408	YHVWWR	2	2	2	R	R	D	R
329	Xinyuanmai 04130	YHVWWR	2	2	2	R	R	R	R
330	Xinnong 518	YHVWWR	2	4	3	R	R	D	R
331	Xingmai 13	YHVWWR	2	2	2	R	R	R	R
332	Xingmai 6	YHVWWR	1	2	2	R	R	R	R
333	Su 553	YHVWWR	1	2	2	R	R	R	R
334	Su 853	YHVWWR	2	2	2	R	R	D	R
335	Xumai 270	YHVWWR	1	2	2	R	R	R	R
336	Xumai 9074	YHVWWR	2	3	2	R	R	R	R
337	Xumai 9158	YHVWWR	2	3	2	R	R	R	R
338	Xuke 1	YHVWWR	2	3	3	R	R	D	R
339	Xuke 718	YHVWWR	2	3	3	R	R	D	R
340	Xumai 1145	YHVWWR	3	3	2	R	R	R	R
341	Xumai 262	YHVWWR	1	2	2	R	R	R	R
342	Xumai 6	YHVWWR	3	4	3	R	D	R	R
343	Yan 0428	YHVWWR	1	2	2	R	R	R	R
344	Yan 2415	YHVWWR	1	2	2	R	R	R	R
345	Yannong 15	YHVWWR	2	2	3	R	R	D	R
346	Yannong 19	YHVWWR	1	1	2	R	R	R	R
347	Yannong 21	YHVWWR	1	1	2	R	R	R	R
348	Yannong 22	YHVWWR	1	2	2	R	R	D	R
349	Yannong 23	YHVWWR	1	2	2	R	R	R	R
350	Yannong 24	YHVWWR	1	2	2	R	R	R	R
351	Yannong 999	YHVWWR	2	2	2	R	R	D	R
352	Yanke 028	MLRYRWWR	3	3	2	R	D	R	R
353	Yanzhan 4110	YHVWWR	2	2	3	R	R	D	R
354	Yangmai 16	MLRYRWWR	3	3	3	R	R	R	R
355	Yangmai 19	MLRYRWWR	3	3	4	R	R	R	R
356	Yangmai 20	MLRYRWWR	3	4	3	R	R	R	R
357	Yangnuomai 1	MLRYRWWR	5	5	5	R	R	D	R
358	Yangguang 828	YHVWWR	2	3	2	R	R	R	R
359	Yangguang 838	SWWWR	1	2	2	R	R	R	R
360	Yangmai 11	MLRYRWWR	3	4	4	R	R	D	R
361	Yangmai 14	MLRYRWWR	3	3	4	R	R	D	R
362	Yangmai 18	MLRYRWWR	3	4	4	R	R	D	R
363	Yi 00-119	MLRYRWWR	5	3	4	R	R	D	R
364	Yimai 11	YHVWWR	2	3	2	R	R	R	R
365	Yikemai 3	YHVWWR	2	2	3	R	R	D	R

366	Yikemai 5	YHVWWR	2	2	2	R	R	D	R
367	Yumai 09113	SWWWR	4	3	3	R	R	D	R
368	Yumai 0926	SWWWR	5	4	5	R	R	D	R
369	Yumai 7	SWWWR	4	4	4	R	R	R	R
370	Yumai 9	SWWWR	4	3	4	R	R	D	R
371	Yuliang 1688	YHVWWR	2	3	2	R	R	D	R
372	Yunong 69	YHVWWR	2	2	2	R	R	D	R
373	Changhe 23	YHVWWR	1	1	2	R	R	R	R
374	Changhe 25	YHVWWR	3	2	2	R	R	R	R
375	Zhenmai 9	YHVWWR	4	4	5	R	R	D	R
376	Zhengmai 9023	YHVWWR	2	3	3	R	R	R	R
377	Zhengmai 113	YHVWWR	2	2	2	R	R	D	R
378	Zhengmai 366	YHVWWR	2	2	2	R	R	D	R
379	Zhengmai 583	YHVWWR	2	3	2	R	R	R	R
380	Zhengmai 883	YHVWWR	2	3	3	R	R	D	R
381	Zhengyumai 0519	YHVWWR	2	2	2	R	R	R	R
382	Zhengyumai 518	YHVWWR	1	2	2	R	R	R	R
383	Zhengyumai 988	YHVWWR	3	3	2	R	R	R	R
384	Zhongfan 4	YHVWWR	3	3	3	R	D	R	R
385	Zhongfan 5	YHVWWR	1	2	2	R	R	R	R
386	Zhongfan 3	YHVWWR	1	1	2	R	R	R	R
387	Zhonghe -108	MLRYRWWR	5	4	5	R	R	D	R
388	Zhonghe -119	MLRYRWWR	3	2	2	R	R	D	R
389	Zhonghe -121	MLRYRWWR	2	2	3	R	R	D	R
390	Zhonghe -123	MLRYRWWR	1	1	2	R	R	D	R
391	Zhonghe -126	MLRYRWWR	3	4	4	R	R	D	R
392	Zhonghe -157	MLRYRWWR	3	3	4	R	R	D	R
393	Zhonghe -159	MLRYRWWR	4	3	3	R	R	D	R
394	Zhonghe -160	MLRYRWWR	3	3	4	R	R	D	R
395	Zhonghe -47	MLRYRWWR	2	3	4	R	R	D	R
396	Zhonghe -57	MLRYRWWR	4	5	4	R	R	D	R
397	Zhonghe -58	MLRYRWWR	2	3	3	R	R	D	R
398	Zhonghe -59	MLRYRWWR	5	5	5	R	R	D	R
399	Zhonghe -6	MLRYRWWR	1	1	3	R	R	D	R
400	Zhonghe -70	MLRYRWWR	3	2	2	R	R	D	R
401	Zhonghe -72	MLRYRWWR	2	3	2	R	R	D	R
402	Zhonghe -75	MLRYRWWR	4	4	4	R	R	D	R
403	Zhongluo 5	YHVWWR	2	3	3	R	R	D	R
404	Zhongluotiegan	YHVWWR	2	3	3	R	R	D	R
405	Zhonmai 1139	NWWR	2	2	3	R	R	D	R
406	Zhonmai 1142	NWWR	2	2	3	R	R	D	R

407	Zhonmai 1143	NWWR	2	3	3	R	R	D	R
408	Zhonmai 1187	NWWR	1	1	2	R	R	R	R
409	Zhonmai 1195	NWWR	1	2	2	R	R	R	R
410	Zhonmai 1196	NWWR	1	2	2	R	R	R	R
411	Zhonmai 14	NWWR	1	2	2	R	R	R	R
412	Zhonmai 895	YHVWWR	3	2	2	R	R	R	R
413	Zhonmai 1140	NWWR	1	2	3	R	R	D	R
414	Zhongyu 1023	YHVWWR	3	4	4	R	R	D	R
415	Zhongyu 1026	YHVWWR	3	3	4	R	R	D	R
416	Zhongyu 1095	YHVWWR	2	2	2	R	R	D	R
417	Zhongyu 1107	YHVWWR	2	2	2	R	R	R	R
418	Zhongyu 1152	YHVWWR	2	3	2	R	R	D	R
419	Zhongyu 1153	YHVWWR	2	3	2	R	R	R	R
420	Zhongyu 9302	YHVWWR	2	3	2	R	R	R	R
421	Zhongyuan 18	YHVWWR	3	3	2	R	R	R	R
422	Zhongzhi 1	YHVWWR	2	2	3	R	R	D	R
423	Zhongzhongmai 10	YHVWWR	2	2	2	R	R	R	R
424	Zhongzhi 9	YHVWWR	1	1	2	R	R	R	R
425	Zhongmai 1	YHVWWR	1	2	2	R	R	R	R
426	Zhongyou 989	YHVWWR	2	1	2	R	R	R	R
427	Zhoumai 22	YHVWWR	2	3	2	R	R	R	R
428	Zhoumai 16	YHVWWR	2	2	2	R	R	R	R
429	Zhoumai 18	YHVWWR	2	3	2	R	R	R	R
430	Zhoumai 24	YHVWWR	2	2	3	R	R	D	R
431	Zhoumai 25	YHVWWR	3	4	4	R	R	R	R
432	Zhoumai 27	YHVWWR	1	2	2	R	R	R	R
433	Zhoumai 31	YHVWWR	2	2	2	R	R	R	R
434	Zhumai 328	YHVWWR	1	1	2	R	R	R	R
435	Zimai 0706	YHVWWR	2	2	2	R	R	R	R

R: recessive allele; D: dominant allele; YHVWWR: the Yellow and Huai Valley Winter Wheat Region; MLRYRWWR: the Middle and Lower Reaches of the Yangtze River Winter Wheat Region; SWWWR: the Southwestern Winter Wheat Region; NWWR: the Northern Winter Wheat Region.

Table S2. The differences in freezing tolerance across different ecological regions.

Ecological Region	Freezing grade (Mean±SD)	Number of accessions
The Northern Winter Wheat Region	1.71±0.52a[1]	13
The Yellow and Huai Valley Winter Wheat Region	2.05±0.60a	268
The Middle and Lower Reaches of the Yangtze River Winter Wheat Region	3.00±0.95b	128
The Southwest Winter Wheat Region	3.32±0.82c	26

1Different lowercase letters indicate significant differences at $P < 0.05$.

Table S3. The jointing-freezing tolerance and vernalizing genotypes of 192 wheat varieties.

No	Accessions	Dead stem rate	Jointing-freezing grade	*Vrn-A1*	*Vrn-B1*	*Vrn-D1*	*Vrn-B3*
1	Aifeng 3	0.25	2	R	R	D	R
2	Aikang 58	0.25	2	R	R	R	R
3	Aizao 78	0.90	5	R	R	D	R
4	Anke 1243	0.23	2	R	R	R	R
5	Anke 1302	0.07	1	R	R	R	R
6	Anke 1303	0.97	5	R	R	R	R
7	Anke 1401	0.81	5	R	R	R	R
8	Anke 157	0.51	4	R	R	R	R
9	Anke 1605	0.33	3	R	R	R	R
10	Anke 1701	0.33	3	R	R	R	R
11	Anke 1702	0.49	4	R	R	R	R
12	Anke 1703	0.83	5	R	R	R	R
13	Anke 1704	1.00	5	R	R	R	R
14	Anke 1706	0.93	5	R	R	R	R
15	Anke 1801	0.81	5	R	R	R	R
16	Anke 1802	0.44	4	R	R	R	R
17	Anke 1803	0.35	3	R	R	R	R
18	Anke 1804	0.41	3	R	R	R	R
19	Anke 1901	0.28	2	R	R	R	R
20	Anke 1902	0.35	3	R	R	R	R
21	Anke 1903	0.30	3	R	R	D	R
22	Anke 1904	0.74	5	R	R	R	R
23	Anke 1907	0.76	5	R	R	R	R
24	Anke 1908	1.00	5	R	R	R	R
25	Anke 2001	0.41	3	R	R	R	R
26	Anke 2002	0.01	1	R	R	R	R
27	Anke 2004	0.34	3	R	R	R	R
28	Anke 2006	0.93	5	R	R	R	R
29	Anke 2007	0.55	4	R	R	R	R
30	Anke 2008	0.64	4	R	R	R	R
31	Anke 2009	0.27	2	R	R	D	R
32	Anke 2101	0.10	1	R	R	R	R
33	Anke 2102	0.51	4	R	R	R	R
34	Anke 2103	0.49	4	R	R	R	R
35	Anke 2201	0.20	2	R	R	R	R
36	Anke 2202	0.54	4	R	R	R	R

37	Anke 2203	0.57	4	R	R	R	R
38	Anke 2208	0.91	5	R	R	D	R
39	Anke 235	0.48	4	R	R	D	R
40	Anke 236	0.54	4	R	R	R	R
41	Anke 237	0.05	1	R	R	R	R
42	Anke 238	0.08	1	R	R	R	R
43	Anke 239	0.10	1	R	R	D	R
44	Annong 0711	0.14	2	R	R	D	R
45	Annong 112	0.14	2	R	R	D	R
46	Bainong 207	0.25	2	R	R	R	R
47	Bainong 3217	0.14	2	R	D	R	R
48	Beijing 8	0.14	2	R	R	R	R
49	Bima 1	0.10	1	R	R	D	R
50	Chuanmai 42	0.18	2	R	R	D	R
51	Fanmai 5	0.90	5	R	R	D	R
52	Fengchan 3	1.00	5	R	R	D	R
53	Fengdecunmai 1	0.06	1	R	R	R	R
54	Fengdecunmai 5	0.04	1	R	R	R	R
55	Gushen 6	0.04	1	R	R	R	R
56	Guinong 775	1.00	5	R	R	R	R
57	Guoshengmai 1	0.97	5	R	R	D	R
58	Han 6172	0.10	1	R	R	D	R
59	Henong 825	0.08	1	R	R	R	R
60	Hengjinmai 8	0.09	1	R	R	D	R
61	Huacheng 2019	0.04	1	R	R	R	R
62	Huacheng 3366	0.10	1	R	R	R	R
63	Huacheng 859	0.98	5	R	R	R	R
64	Huacheng 863	0.23	2	R	R	D	R
65	Huaimai 18	0.93	5	R	R	D	R
66	Huaimai 20	0.61	4	R	R	R	R
67	Huaimai 22	0.11	1	R	R	D	R
68	Huaimai 25	0.98	5	R	R	R	R
69	Huaimai 29	0.19	2	R	R	R	R
70	Huaimai 30	0.19	2	R	D	R	R
71	Huaimai 33	0.90	5	R	R	D	R
72	Huaimai 39	0.87	5	R	R	D	R
73	Huaimai 40	0.31	3	R	R	D	R
74	Huaimai 44	0.77	5	R	R	R	R
75	Huaimai 45	0.61	4	R	R	R	R
76	Huaimai 920	0.96	5	R	R	R	R

77	Huiyan 22	0.20	2	R	R	R	R
78	Huiyan 66	0.90	5	R	R	R	R
79	Huiyan 77	0.88	5	R	R	R	R
80	Huiyan 912	0.96	5	R	R	R	R
81	Jimai 20	0.89	5	R	R	R	R
82	Jimai 22	0.92	5	R	R	R	R
83	Jimai 44	0.22	2	R	R	R	R
84	Jinan 13	0.19	2	R	R	R	R
85	Jinan 17	0.95	5	R	R	R	R
86	Jinan 2	0.93	5	R	R	R	R
87	Jimai 38	0.90	5	R	R	R	R
88	jinmai 33	0.96	5	R	R	R	R
89	Jinmai 45	0.30	3	R	R	R	R
90	Jinmai 73	0.51	4	R	R	R	R
91	Kaimai 18	0.94	5	R	R	R	R
92	Lemai 207	0.88	5	R	R	R	R
93	Ligao 6	1.00	5	R	R	R	R
94	Lianmai 2	0.41	3	R	R	D	R
95	Liangxing 99	0.96	5	R	R	R	R
96	Liumai 618	0.19	2	R	R	D	R
97	Longke 1109	0.95	5	R	R	D	R
98	Lumai 14	0.96	5	R	R	R	R
99	Lumai 15	0.96	5	R	R	D	R
100	Lumai 1	0.73	5	R	R	R	R
101	Lumai 21	0.71	5	R	R	R	R
102	Lumai 7	1.00	5	R	R	D	R
103	Luyuan 502	0.52	4	R	R	R	R
104	Lunxuan 988	0.68	5	R	R	D	R
105	Luomai 21	0.63	4	R	R	D	R
106	Luomai 26	0.60	4	R	R	D	R
107	Luomai 29	0.91	5	R	R	R	R
108	Mianmai 39	0.97	5	R	R	D	R
109	Mingmai 1	0.96	5	R	R	D	R
110	Nanda 2419	1.00	5	R	R	D	R
111	Nannong 0686	0.25	2	R	R	D	R
112	Niemai 836	1.00	5	R	R	R	R
113	Neimai 8	0.20	2	R	R	D	R
114	Ningmai 13	0.28	2	R	R	D	R
115	Ningmai 18	0.53	4	R	R	D	R
116	Ningmai 24	0.67	5	R	R	D	R

117	Qianmai 18	0.74	5	R	R	R	R
118	Qingnong 3	0.44	4	R	R	R	R
119	Quanmai 725	0.97	5	R	R	R	R
120	Ruihuamai 516	0.93	5	R	R	D	R
121	Ruihuamai 518	0.93	5	R	R	D	R
122	Ruihuamai 520	0.88	5	R	R	D	R
123	Shannong 17	0.22	2	R	R	R	R
124	Shannong 20	0.37	3	R	R	R	R
125	Han 7859	0.33	3	R	D	D	R
126	Shengxuan 6	0.82	5	R	R	D	R
127	Shijiazhuang 4185	0.59	4	R	R	D	R
128	Shijiazhuang 407	0.75	5	R	R	R	R
129	Shijiazhuang 54	0.61	4	R	R	D	R
130	Shijiazhuang 8	0.78	5	R	R	R	R
131	Shite 14	1.00	5	R	R	D	R
132	Tainong 19	0.75	5	R	R	R	R
133	Tianyike 5	0.28	2	R	R	D	R
134	Wan 1204	0.64	4	R	R	R	R
135	Wankenmai 0622	1.00	5	R	R	D	R
136	Wanmai 32	0.40	3	R	R	D	R
137	Wanmai 38	0.52	4	R	R	R	R
138	Wanmai 47	1.00	5	R	R	R	R
139	Wanmai 50	0.92	5	R	R	R	R
140	Wanmai 52	1.00	5	R	R	R	R
141	Wanmai 53	0.93	5	R	R	R	R
142	Wanmai 606	0.88	5	R	R	D	R
143	Weilai 0818	1.00	5	R	R	R	R
144	Guomai 11	0.88	5	R	R	D	R
145	Guomai 182	0.91	5	R	R	R	R
146	Guomai 8	0.86	5	R	R	R	R
147	Guomai 99	0.92	5	R	R	D	R
148	Guomai 9	0.63	4	R	R	R	R
149	Xian 8	0.90	5	R	R	D	R
150	Xinong 511	0.92	5	R	R	R	R
151	Xinong 6028	1.00	5	R	R	D	R
152	Xinong 822	0.75	5	R	R	R	R
153	Xinong 889	0.69	5	R	R	R	R
154	Xiaoyan 6	0.11	1	R	R	R	R
155	Xinmai 18	0.89	5	R	R	R	R
156	Xinmai 26	0.92	5	R	R	R	R

157	Su 553	0.43	4	R	R	R	R
158	Xunong 029	0.37	3	R	R	D	R
159	Xuzhou 14	0.94	5	R	R	R	R
160	Yannong 19	1.00	5	R	R	R	R
161	Yannong 21	1.00	5	R	R	R	R
162	Yannong 5158	0.28	2	R	R	R	R
163	Yannong 999	0.55	4	R	R	D	R
164	Yanzhan 4110	0.51	4	R	R	D	R
165	Yangfumai 4	0.21	2	R	R	D	R
166	Yangmai 158	0.99	5	R	R	D	R
167	Yangmai 20	0.95	5	R	R	D	R
168	Yangmai 22	0.93	5	R	R	R	R
169	Yangmai 23	0.41	3	R	R	D	R
170	Yangmai 24	0.44	4	R	R	R	R
171	Yangmai25	0.80	5	R	R	R	R
172	Yangnuomai 1	0.41	3	R	R	D	R
173	Yumai 13	0.53	4	R	R	D	R
174	Yumai 18	0.74	5	R	R	D	R
175	Yumai 21	1.00	5	R	R	R	R
176	Yumai 2	0.63	4	R	R	D	R
177	Yumai 49	0.79	5	R	R	R	R
178	Yumai 7	0.53	4	R	R	D	R
179	Zhengmai 7698	1.00	5	R	R	R	R
180	Zhengmai 366	0.42	3	R	R	R	R
181	Zhengmai 9023	0.36	3	R	R	R	R
182	Zhengzhou 8329	0.93	5	R	R	D	R
183	Zhongmai 578	0.97	5	R	R	R	R
184	Zhongmai 895	0.81	5	R	R	D	R
185	Zhoumai 18	0.53	4	R	R	R	R
186	Zhoumai 22	0.40	3	R	R	R	R
187	Zhoumai 26	0.53	4	R	R	R	R
188	Zhoumai 28	0.56	4	R	R	D	R
189	Zhoumai 30	0.40	3	R	R	D	R
190	Zhoumai 32	0.38	3	R	R	R	R
191	Zhoumai 36	0.50	4	R	R	R	R
192	Zimai 19	0.41	3	R	R	D	R

R: recessive allele; D: dominant allele

Table S4. Primer sequences used to identify *VRN-1* and *VRN-B3* alleles.

Gene name	Marker name	Primer sequence （5'-3'）	Allelic variation	Allelic variation (bp)	Annealing temperature (℃)	Extension time (s)
VRN-A1	*Vrn1-AF*	GAAAGGAAAAATTCTGCTCG	*Vrn-A1a*	965+876	50	60
	Vrn1-Int1R	GCAGGAAATCGAAATCGAAG	*Vrn-A1b*	714		
			Vrn-A1c	734		
			vrn-A1	734		
	Intr1-AF2	AGCCTCCACGGTTTGAAAGTAA	*Vrn-A1c*	1170	56	65
	Intr1-AR3	AAGTAAGACAACAC GAATGT GAGA				
	Intr1-CF	GCACTCCTAACCCACTAACC	*vrn-A1*	1068	58	65
	Intr1-ABR	TCATCCATCATCAAGGCAAA				
VRN-B1	*Intr-BF*	CAAGTGGAACGGTTAGGACA	*Vrn-B1*	709	63	43
	Intr1-BR3	CTCATGCCAAAAATTGAAGATGA				
	Intr1-BR4	CAAATGAAAAGGAATGAGAGCA	*vrn-B1*	1149	58	69
VRN-D1	*Intr1-DF*	GTTGTCTGCCTCATCAAATCC	*Vrn-D1*	1671	65	90
